# Iodixanol density gradients as an effective phytoplasma enrichment approach to improve genome sequencing

**DOI:** 10.3389/fmicb.2022.937648

**Published:** 2022-08-12

**Authors:** Bianca Rodrigues Jardim, Lucy T. T. Tran-Nguyen, Cherie Gambley, Brendan Rodoni, Fiona E. Constable

**Affiliations:** ^1^School of Applied Systems Biology, La Trobe University, Bundoora, VIC, Australia; ^2^Agriculture Victoria Research, Department of Jobs, Precincts and Regions, AgriBio Centre, Bundoora, VIC, Australia; ^3^Plant Health Australia, Deakin, ACT, Australia; ^4^Horticulture and Forestry Science, Department of Agriculture and Fisheries, Maroochy Research Facility, Nambour, QLD, Australia

**Keywords:** host DNA contamination, unculturable bacteria, phytopathogen, natural host, OptiprepTM, density gradient centrifugation, high-throughput sequencing, 16SrII phytoplasma

## Abstract

Obtaining complete phytoplasma genomes is difficult due to the lack of a culture system for these bacteria. To improve genome assembly, a non-ionic, low- and iso-osmotic iodixanol (Optiprep™) density gradient centrifugation method was developed to enrich for phytoplasma cells and deplete plant host tissues prior to deoxyribonucleic acid (DNA) extraction and high-throughput sequencing (HTS). After density gradient enrichment, potato infected with a ‘*Candidatus* Phytoplasma australasia’-related strain showed a ∼14-fold increase in phytoplasma HTS reads, with a ∼1.7-fold decrease in host genomic reads compared to the DNA extracted from the same sample without density gradient centrifugation enrichment. Additionally, phytoplasma genome assemblies from libraries equalized to 5 million reads were, on average, ∼15,000 bp larger and more contiguous (N50 ∼14,800 bp larger) than assemblies from the DNA extracted from the infected potato without enrichment. The method was repeated on capsicum infected with Sweet Potato Little Leaf phytoplasma (‘*Ca.* Phytoplasma australasia’-related strain) with a lower phytoplasma titer than the potato. In capsicum, ∼threefold more phytoplasma reads and ∼twofold less host genomic reads were obtained, with the genome assembly size and N50 values from libraries equalized to 3.4 million reads ∼137,000 and ∼4,000 bp larger, respectively, compared to the DNA extracted from infected capsicum without enrichment. Phytoplasmas from potato and capsicum were both enriched at a density of 1.049–1.058 g/ml. Finally, we present two highly contiguous ‘*Ca.* Phytoplasma australasia’ phytoplasma reference genomes sequenced from naturally infected *Solanaceae* hosts in Australia. Obtaining high-quality phytoplasma genomes from naturally infected hosts will improve insights into phytoplasma taxonomy, which will improve their detection and disease management.

## Introduction

Phytoplasmas (class Mollicutes) are unculturable, insect-vectored phytopathogens of more than 700 economically important plants (Trivellone and Dietrich, 2021). They lack cell walls and reside intracellularly in their insect and plant hosts (phloem limited). Phytoplasmas are associated with disrupted development of stem, leaf, or fruiting tissues and, thereby, reduce the yield or survival of the infected plant. Whole genome sequences are central to our understanding of phytoplasma symptomology and metabolic properties, their evolution and taxonomy, epidemiology, and can improve diagnosis (Oshima et al., 2004; Sugio et al., 2011; Chung et al., 2013; Orlovskis et al., 2017; Cho et al., 2020; Zhao et al., 2021). However, obtaining complete phytoplasma genomes is difficult because they are yet to be cultured and their low titer in most plant hosts limits the amount of phytoplasma chromosomes obtained from total deoxyribonucleic acid (DNA) extractions. Additionally, phytoplasma genomes are AT-rich (generally over 70% AT) and contain many duplicated genes, which makes assembly from widely used short-read sequencing data a further challenge (Oyola et al., 2012).

Recovering phytoplasma genomes directly from an infected host remains a major technical challenge due to the high proportion of “contaminating” host genomic and organellar DNA (mitochondrial DNA, plastid DNA) compared to phytoplasma DNA (Tran-Nguyen and Gibb, 2007). Phytoplasma genomes are also many magnitudes smaller than the host genome and direct sequencing of these samples reduces the depth to which phytoplasma-derived DNA is sequenced. It is therefore inefficient and costly to close sequence gaps in the target phytoplasma genomes when a high proportion of host DNA is present in the DNA extract. While genome assembly algorithms are improving to recover complete or draft metagenomic-assembled genomes of a high accuracy and quality, there is still a need to use and develop procedures to deplete host DNA and/or enrich for microbial DNA (Pereira-Marques et al., 2019), especially for unculturable phytopathogens such as phytoplasmas.

To date, only three complete phytoplasma genomes have been obtained without enrichment procedures (Orlovskis et al., 2017; Coetzee et al., 2019; Huang et al., 2022), and most others were obtained using the post-DNA extraction enrichment approaches, such as cesium chloride (CsCl) density gradient centrifugation and/or pulsed-field gel electrophoresis (PFGE) (Oshima et al., 2004; Bai et al., 2006; Kube et al., 2008; Tran-Nguyen et al., 2008; Andersen et al., 2013; Wang et al., 2018). Many draft genomes have been assembled from DNA extractions from original phytoplasma-infected hosts or artificially infected high-titer hosts with (Chen et al., 2014; Kakizawa et al., 2014; Fischer et al., 2016; Music et al., 2019) or without enrichment procedures (Chung et al., 2013). However, some of these methods require several days of wet lab processing, multiple sets of specialized equipment, the use of harsh chemicals, and are only effective when the phytoplasma titer in the host plant is high; for example, the method implementing CsCl density gradient centrifugation followed by PFGE.

A phytoplasma enrichment method that is cost-effective, requires minimal specialized equipment, shorter processing times, and that does not rely on transmission of the phytoplasma to high-titer hosts for the method to be effective will advance our current understanding of phytoplasma genomics. Ideally, the enrichment method should be applied either prior to or after nucleic acid extraction but before sequencing to reduce the sequencing cost and to improve the accuracy of assembled genomes from metagenomic datasets. Recently, a kit-based post-DNA extraction method was used to deplete methylated host DNA directly from greenhouse-maintained (Nijo et al., 2021) or field-collected (Kirdat et al., 2021) plants infected with phytoplasmas. A more phytoplasma-targeted enrichment method was also developed based on the immunodominant membrane proteins (IMPs) in phytoplasma membranes (Tan et al., 2021). Here, we report the development of an approach using iodixanol (Optiprep™, Sigma–Aldrich, Missouri, MI, United States), a clinical-grade, non-ionic, and iso-osmotic density gradient medium, to enrich for phytoplasma cells directly from two naturally infected hosts prior to the DNA extraction. Using quantitative polymerase chain reaction (qPCR) and reference mapping of metagenomic reads, we demonstrate that the method reduces the proportion of host-derived sequences and increases phytoplasma sequences in the DNA extract; thus, improving the quality and completeness of the metagenomic-assembled phytoplasma genomes, particularly for ‘*Ca.* Phytoplasma australasia’-related phytoplasmas infecting *Solanaceae* species. The resultant draft phytoplasma genome assemblies of the infected potato and capsicum were used in comparative genomic analyses to illustrate that the method produces genomes with a suitable completeness to infer taxonomic relationships between taxa.

## Materials and methods

### Plant material

The plant samples used in this study are listed in Table 1. The potato sample was propagated and maintained under natural daylight and day length conditions at ambient temperature in a screenhouse, with a regular watering and in an insect–proof cage. The potato was maintained in the screenhouse for approximately 15 months from initial propagation to sampling. The capsicum field sample was processed directly without propagation.

**TABLE 1:** Sample name, host information, collection location in Australia of samples positive for phytoplasma used in the study as well as the phytoplasma identity based on the top BLASTn hit of the R16F2n/m23sr amplicon and species assignment.

Sample name	Host common name (species name)	Host family	Sampling location in Australia	Highest percent identity (accession number)	BLASTn top hit description
o4P	Potato (*Solanum tuberosum* cv Nadine)	Solanaceae	Melbourne, VIC	99.94% (Y10096)	‘*Ca.* Phytoplasma australasia’
o7C	Capsicum (*Capsicum annuum*)	Solanaceae	Stanthorpe, QLD	99.87% (JQ868446)	Sweet Potato Little Leaf phytoplasma

### Polymerase chain reaction-based phytoplasma detection and identification

To confirm infection and determine the phytoplasma species present in the samples, total DNA was extracted, PCR amplified, and Sanger sequenced (Macrogen Korea) as stated in Rodrigues Jardim et al. (2021). The identity of the sequenced amplicon was confirmed by BLASTn analysis (Altschul et al., 1997) of the approximately 1,600 base pair (bp) sequence.

### Estimation of phytoplasma titer and concentration using quantitative polymerase chain reaction

To estimate the efficiency of the qPCR reaction and, hence, accurately monitor the presence of phytoplasma, qualitatively and quantitatively, in the DNA extracts of samples collected prior to enrichment, after the differential centrifugation, and after the density gradient centrifugation, a standard curve was produced using a plasmid ligated with the R16F2n/m23sr-nested PCR amplicon of the phytoplasma-infected potato sample used in this study. The amplicon was cloned, purified, and Sanger sequenced as previously described (Rodrigues Jardim et al., 2021) to confirm the identity of the insert DNA. Plasmids ligated with the R16F2n/m23sr amplicon were extracted using the QIAprep Spin Miniprep Kit (Qiagen) following the manufacturer’s specifications. The extracted plasmid DNA concentration was measured using a NanoDrop™ spectrophotometer. To produce a qPCR standard curve with the phytoplasma 16S rRNA gene sequence as the target, the ligated plasmids were serially diluted 10-fold with UltraPure™ DNase/RNase-Free Distilled Water (Thermo Fisher Scientific, Massachusetts, MA, United States) for a total of eight dilution points.

The GoTaq^®^ Probe 1-Step qPCR System (Promega Wisconsin, WI, United States) was used for qPCR according to the manufacturer’s instructions for a total reaction volume of 20 μl. The phytoplasma 16S rRNA gene specific primers and TaqMan probes described by Christensen et al. (2004) were used at published concentrations. Four microliters of template DNA from each sample or each plasmid dilution were used per qPCR reaction. Four replicates of each plasmid serial dilution were included in every qPCR 96- or 384-well plate for absolute quantification of phytoplasma DNA, as well as four replicates of a template-free control to monitor for contamination, and three replicates from each sample were included. All qPCRs were set up and run on a QuantStudio™ 3 Real-Time PCR System (Thermo Fisher Scientific, Massachusetts, MA, United States) with analysis performed using the QuantStudio™ Design and Analysis Software (Thermo Fisher Scientific, Massachusetts, MA, United States). The qPCR efficiencies and *R*^2^ values per run were estimated using the QuantStudio™ Design and Analysis Software (Thermo Fisher Scientific, Massachusetts, MA, United States).

### Sample preparation for iodixanol density gradient centrifugation

Supplementary Appendix 1 contains a detailed step-by-step protocol for sample preparation, differential centrifugation, and iodixanol density gradient centrifugation with specifications for three technical replicates per host and one negative control per run (see also Supplementary Appendix Figure 1), iodixanol fraction sampling (Supplementary Appendix Figure 2), and a DNA extraction protocol for all sample types. The details of Supplementary Appendix 1 are summarized with the appropriate references.

#### Tissue sampling

Potato tissues with typical phytoplasma symptoms were collected from the screenhouse and processed on the day of collection. The symptomatic capsicum sample was transported at ambient temperature for approximately 3 days from the sampling location (Table 1) to the laboratory and then stored at 4°C upon arrival and until use. The capsicum sample was processed within 5 days of sampling to minimize the impact of degradation on the intactness of phytoplasma cells. Whole leaves, petioles, and stems of the potato and capsicum samples were used.

#### Sample preparation and homogenization

To homogenize the samples, 16 g of plant material was added to an extraction bag (BIOREBA Kanton Reinach, Switzerland) with 210-ml ice-cold Phytoplasma Grinding Buffer (pH 7.6 at 4°C; Palmano, 2001). All samples were then gently homogenized using the semi-automated HOMEX 6 (BIOREBA Kanton Reinach, Switzerland) set at a low speed (10% of the maximum speed). The homogenates were refrigerated at 4°C for 20 min to allow all cells to plasmolyze.

#### Differential centrifugation

After plasmolysis, the tissue homogenates were split into two 48 ml aliquots in ice-cold 50-ml conical screw cap tubes and centrifuged at a low speed (1,500 rcf) for 10 min at 4°C in a fixed-angle rotor (F-3-6-38 rotor; Eppendorf) to remove cellular and environmental debris from the sample. The resultant supernatants were then transferred to new ice-cold 50-ml conical screw cap tubes and centrifuged at a high speed (10,000 rcf) for 30 min at 4°C in the same fixed-angle rotor to pellet intact cells. The resultant pellets from the first high-speed centrifugation were gently resuspended in 40 ml Tris-Sucrose-EDTA buffer (TSE, pH 8 at 4°C; Quan et al., 2013) and differentially centrifuged as above, with the exception that the resultant high-speed centrifugation pellets were each resuspended in 800-μl TSE, pooled into a single 50-ml conical screw cap tube (referred to as “differentially centrifuged pellet” or abbreviated with the suffix “TSE” in tables and figures), and kept at 4°C until use. The samples collected for DNA extraction and subsequent phytoplasma quantification by qPCR and high-throughput sequencing (HTS) included approximately 400 μl each of unprocessed tissue homogenate (no differential centrifugation) and the final pellet after differential centrifugation (resuspended in 4 ml of CTAB extraction buffer). Each sample was reserved in a separate 2-ml capped centrifuge tube and stored at –20°C until required.

#### Iodixanol density gradient centrifugation

A 40% (v/v) iodixanol (Optiprep™, Sigma–Aldrich, Missouri, MI, United States) working solution was prepared by diluting the 60% iodixanol stock solution with the diluent solution (0.15-M NaCl, 1-mM EDTA, 15-mM Tris; pH 7.6 at 4°C; Frampton et al., 2018). Three subsequent iodixanol solutions [14, 22, and 30% (v/v)] were prepared by diluting the 40% (v/v) iodixanol working solution in a NaCl-EDTA-Tris buffer (0.15-M NaCl, 0.5-mM EDTA, 5-mM Tris; pH 7.6 at 4°C; Frampton et al., 2018). The density gradient was prepared on ice and by first adding 3 ml 30% (v/v) iodixanol to a 13.2-ml open–top thin wall polypropylene centrifuge tube (Beckman Coulter Life Sciences, California, CA, United States) using a 20 gauge needle (Terumo Medical Corporation, Tokyo, Japan) and a 20-ml syringe, followed by gently adding 4 ml of the 22% (v/v) iodixanol solution, and 4 ml of the 14% (v/v) iodixanol. Three technical replicates were prepared and each gradient was overlaid with 400 μl of the final TSE cell suspension immediately after they were prepared. An additional gradient was used as a negative iodixanol control in every run and was overlaid with 400-μl TSE buffer (cell-free). All tubes were balanced prior to centrifugation with cell-free TSE buffer. After the addition of the samples or control, the gradients were centrifuged in a pre-cooled Beckman Coulter Optima L-100 XP ultracentrifuge (SW 41 Ti rotor; 80,000 rcf, Beckman Coulter Life Sciences, California, CA, United States) for 3 h at 4°C. Seven 1.4-ml fractions (Fractions 1–7) and one approximately 2-ml fraction (Fraction 8) of the gradients were consecutively sampled from the top to the bottom of the centrifuge tube, resulting in eight fractions per replicate. The fractions were frozen at –20°C until needed for the DNA extraction. The density in g/ml of each fraction (Supplementary Table 1) was estimated prior to the DNA extraction by weighing the sampled fraction to three decimal places using a Pioneer™ Plus analytical balance (Model PA214C, Ohaus^®^).

#### Deoxyribonucleic acid extraction, quantification, and quality measures of unprocessed, differentially centrifuged, and density gradient samples

Total DNA was extracted from 400 μl of unprocessed homogenate or resuspended pellet that resulted after two rounds of differential centrifugation, and 1.4–2 ml of the density gradient fractions using a modified CTAB and chloroform:isoamyl alcohol (24:1) method (Zhang et al., 1998). The modifications to the DNA extraction method included using 5 ml each of CTAB and chloroform:isoamyl alcohol (24:1) (Sigma–Aldrich, Missouri, MI, United States) per sample, and incubation with isopropanol was done overnight at –20°C to precipitate the DNA. All DNA extractions were performed in 15-ml conical screw cap tubes and were centrifuged in a swinging bucket rotor (A-4-44 rotor, Eppendorf). During the chloroform:isoamyl alcohol (24:1) partitioning step, the samples were centrifuged at 3,500 rcf for 5 min at room temperature. This partitioning step was repeated 2–3 times per sample by adding 5 ml of chloroform:isoamyl alcohol (24:1) until the white interface was absent. The samples were centrifuged at 4,500 rcf for 20 min at 4°C after overnight precipitation in isopropanol (same volume as sample) and the pelleted DNA washed twice with 5 ml of 75% ethanol by centrifugation at 3,500 rcf for 15 min at 4°C. The DNA was resuspended in 60-μl UltraPure™ DNase/RNase-Free Distilled Water (Thermo Fisher Scientific, Massachusetts, MA, United States). A non-template (negative) DNA extraction control was performed alongside extractions to monitor for contamination during the DNA extraction and within the reagents. All DNA extractions were stored at –20°C until use. The DNA quantity (Supplementary Table 2) was estimated using the Qubit™ 1X dsDNA HS Assay Kit (Thermo Fisher Scientific, Massachusetts, MA, United States) on a Qubit™ 2.0 fluorometer (Thermo Fisher Scientific, Massachusetts, MA, United States).

#### Deoxyribonucleic acid sequencing library preparation and Illumina high-throughput sequencing

Library preparation was performed on DNA of the unprocessed plant homogenates, differentially centrifuged pellets, and density gradient samples using the NEXTFLEX^®^ Rapid XP DNA-Seq Kit (PerkinElmer, Massachusetts, MA, United States) with the NEXTFLEX 384 Unique Dual Index Barcodes version 19.06 (PerkinElmer, Massachusetts, MA, United States). The protocol for library preparation without size selection and for inputs of 1 ng specified by the NEXTFLEX Rapid XP DNA-Seq Kit was followed for all samples. All replicates were sequenced for iodixanol fractions that showed the highest concentration of phytoplasma DNA as determined by qPCR screening, while only one replicate was sequenced for the remaining iodixanol fractions to determine their phytoplasma enrichment profiles (Supplementary Table 2). Since the DNA extractions from iodixanol fractions generally had a low DNA concentration (<0.005–1.05 ng/μl based on the Qubit™ fluorometer; Supplementary Table 2), the same volumes of the extracted DNA (34 μl) were used as inputs for library preparation to maximize the data output for each. The input DNA of the unprocessed homogenates and the differentially centrifuged pellets were diluted to 1-ng input DNA to apply the same library preparation protocol as the low DNA concentration iodixanol samples. The input DNA was fragmented according to the manufacturer’s protocol to generate libraries with fragment sizes between 300 and 400 bp. The concentration and fragment sizes of the final libraries were estimated using an Agilent 2200 TapeStation System with the HSD1000 ScreenTape assay (Agilent Technologies, California, CA, United States). The resultant libraries were size-selected using ProNex^®^ Size Selective Purification System (Promega Wisconsin, WI, United States), pooled together in equimolar concentrations, and sequenced on an Illumina NovaSeq 6000 System with an SP Reagent Kit v1.5 (2 × 250 bp).

### Bioinformatic analyses

#### Illumina reads quality control

FastP (Chen et al., 2018), with the specified parameters -3 -5 -q 20 -l 50, was used to trim adapters and low-quality sequences, and to discard reads shorter than 50 bp from the sequence data. To enable a fair comparison between all sequenced samples, the number of trimmed reads per library were randomly down-sampled using Seqtk-1.3 (r106)^1^ to achieve equal sequencing depths. All libraries of interest for the potato and capsicum samples were down-sampled to contain 5,000,000 and 3,400,000 reads/library, respectively. The negative control libraries were not randomly down-sampled to enable the effective detection of any contamination but also due to their low total read numbers after sequencing.

#### Metagenome assemblies and assembly assessments

To obtain a high-quality reference genome and plasmid assemblies of the phytoplasma infecting the potato and capsicum samples investigated in the study, all trimmed reads without down-sampling were *de novo* assembled using SPAdes 3.15.2 (Prjibelski et al., 2020) with the following parameters: –meta, -k 21,33,55,77,99,127 (Nurk et al., 2017). Putative phytoplasma-derived genome and plasmid contigs were identified by BLASTn searches against the NCBI non-redundant database (acquired on January 1, 2022) using BLAST + v2.11.0 (Altschul et al., 1997). Genome sizes and GC content were estimated using Geneious prime 2022.1.1.^2^ The genome N50 values of these draft genome assemblies were evaluated using metaQUAST (Mikheenko et al., 2016). Phytoplasma-derived protein-coding, tRNA, and rRNA genes were annotated and counted using Prodigal (Hyatt et al., 2010), ARAGORN (Laslett and Canback, 2004), and RNAmmer (Lagesen et al., 2007), respectively, implemented in Prokka 1.14.5 (Seemann, 2014). The coverage of the assembled phytoplasma genomes was estimated using BBSplit implemented in the BBMap v.38.61b software suite (Bushnell, 2014) using the assembled phytoplasma as its reference to which the corresponding reads were mapped. Of all these assemblies, one phytoplasma genome per host was selected as the reference phytoplasma genome for the potato and capsicum samples when the genome (i) had the largest N50 value of all assemblies, (ii) encoded a similar number tRNA genes as other publicly available phytoplasma genomes from closely related phytoplasma taxa, (iii) had the highest read coverage, and (iv) had less than 50 phytoplasma-specific contigs above 1,000 bp in size. The *secA* and *tufB* genes were extracted from each chosen reference genome and used in gene alignments with those of ‘*Ca.* Phytoplasma australasia’ (GenBank accession numbers EU168728 and JQ824250, respectively) and ‘*Ca.* Phytoplasma aurantifolia’ (GenBank accession numbers EU168731 and JQ824276, respectively) to determine the phytoplasma species from which the genome arises according to guidelines by Bertaccini et al. (2022).

For the fair assessments on the completeness and contiguity of phytoplasma genomes assembled from samples that underwent iodixanol-based density gradient centrifugation and those that did not, the down-sampled reads were also used in *de novo* assemblies (referred to as down-sampled assemblies) using the same SPAdes and BLASTn parameters discussed above. Additionally, the same genome annotation and quality assessments were performed for the down-sampled genome assemblies as those performed for assemblies without read down-sampling.

#### Read mapping pipeline and taxonomic classification of unmapped reads

Trimmed reads and the randomly down-sampled trimmed reads were mapped to reference genomes (publicly available and assembled in this study, Table 2) using BBSplit 38.61 from the BBMap v.38.61b software suite (Bushnell, 2014) to estimate the relative proportion of phytoplasma DNA, host genomic DNA (gDNA), host mitochondrial DNA (mtDNA), and host chloroplast DNA (cpDNA) per library. The default parameters were used and “ambig2=best” specified to assign the reads to the best-mapped site. The reference genomes specified for BBSplit 38.61 were downloaded from the NCBI (last accessed May 2022) and included, where available, host genomic DNA (potato: GCF_000226075.1; capsicum: GCF_000710875.1), host chloroplast genome (potato: NC_008096.2; capsicum: NC_018552.1), host mitochondrial genome (potato: NC_059127.1; capsicum: NC_024624.1), and two phytoplasma genomes of closely related phytoplasma taxa, as well as the reference genomes assembled in this study (Table 2).

**TABLE 2:** Assembly details and statistics for phytoplasma genomes used as references in this study, including GenBank accession numbers, closest phytoplasma relative, sequencing details where available (tissue sampled, enrichment method, sequencing platforms, and sequencing depths), genome properties, plasmid accession number/name.

Sample name (accession number)	NCHU2014 (GCA_001307505.2)	PR08 (GCA_015239935.3)	o4P (JALQCT000000000)^A^	o7C (JALQCV000000000)^A^
Putative phytoplasma species	‘*Ca.* Phytoplasma aurantifolia’-related strain	‘*Ca.* Phytoplasma australasia’-related strain	‘*Ca.* Phytoplasma australasia’-related strain	SPLL ‘*Ca.* Phytoplasma australasia’-related strain
Host tissue sampled	*Catharanthus roseus* (periwinkle) mature leaves	*Parthenium hysterophorus* leaves	*Solanum tuberosum* (potato) leaves, petioles, and stems	*Capsicum annuum* (capsicum) leaves, petioles, and stems
Enrichment method	Transmission to periwinkle by dodder, and immunoprecipitation	NA	Differential centrifugation and iodixanol density gradient centrifugation, iodixanol Fraction 2 from replicate 2	Differential centrifugation and iodixanol density gradient centrifugation, iodixanol Fraction 7 from replicate 1
Sequencing platform(s)	Illumina and Oxford Nanopore Technology	Illumina and Oxford Nanopore Technology	Illumina	Illumina
Amount of HTS data used (bp)	Approx. 0.67 × 10^9^ (Oxford Nanopore Technology) with approx. 17.16 × 10^9^ (Illumina)	NA	5.94 × 10^9^	19.14 × 10^9^
No. of contigs	1	1	30	29
Est. genome size (bp)	635,584	588,746	555,927	555,321
N50 value (bp)	635,584	588,746	41,668	41,719
Est. genome coverage (×)	2117	2757.98	1372	117
GC (%)	24.5	24.3	23.9	23.7
No. protein coding genes	471	468	463	458
No. tRNAs	24	27	25	25
No. 16S rRNAs	2	2	1	1
Plasmid recovered? (accession number)	Yes (CP040926.1)	No	Putative plasmid: po4P16SrIID	Putative plasmid: po7C16SrIID
References	Chang et al., 2015; Tan et al., 2021	NA	This study	This study

NA, not available.

*^A^secA* and *tufB* gene regions extracted from the genome assemblies of strain o4 and strain o7 share 100% nucleotide sequence identity to those of ‘Ca. Phytoplasma australasia’ (GenBank accession numbers EU168728 and JQ824250, respectively) and only 91–94% with the genes of ‘Ca. Phytoplasma aurantifolia’ (GenBank accession numbers EU168731 and JQ824276, respectively). This places both strains as ‘Ca. Phytoplasma australasia’-related species based on recommendations by Bertaccini et al. (2022).

Unmapped reads from the libraries (not down-sampled) were taxonomically classified using k-mer-based classification with Kraken2 using the abfvhp (archaea, bacteria, fungi, virus, human, and plasmid) genome database (Wood et al., 2019). Kraken2 reports were visualized in Pavian (Breitwieser and Salzberg, 2020).

#### Whole genome comparisons

Publicly available phytoplasma genomes and the phytoplasma reference genomes assembled in this study from potato and capsicum hosts were compared with phytoplasma genomes assembled from down-sampled reads from the unprocessed tissue homogenates, differentially centrifuged pellets, and iodixanol fraction with the highest proportion of phytoplasma HTS reads for the infected potato and capsicum hosts (Table 2). The average nucleotide identities (ANIs) between the genomes were calculated using FastANI version 1.33 (Jain et al., 2018), and the proportion of mapped genomic segments were calculated according to Cho et al. (2020) per sample.

## Results

### Phytoplasma screening and identification by polymerase chain reaction and Sanger sequencing

BLASTn analysis of the directly Sanger sequenced R16F2n/m23sr nested PCR amplicon confirmed the infection of a ‘*Candidatus* Phytoplasma australasia’ phytoplasma in the potato sample and a Sweet Potato Little Leaf (SPLL) phytoplasma in the capsicum sample (Table 1).

### Iodixanol fraction densities after centrifugation

After centrifugation, the density of the sampled fractions ranged between 1.049 (Fraction 1) and 1.196 g/ml (Fraction 8) for the potato replicates, and from 1.058 (Fraction 1) to 1.157 g/ml (Fraction 8) for the capsicum replicates (Supplementary Table 1).

### Deoxyribonucleic acid concentration estimates before density gradient centrifugation, and across all fractions after density gradient centrifugation

DNA concentrations of unprocessed homogenates for each sample were all above 20 ng/μl (Table 3 and Supplementary Table 2). When compared to the DNA extracted from the same volume of the differentially centrifuged pellets, the unprocessed homogenates showed a 20-fold higher concentration in the case of the potato samples, and an almost fourfold higher DNA concentration for the capsicum sample.

**TABLE 3:** Summary of iodixanol fraction densities after gradient centrifugation of phytoplasma infected potato (o4P) and capsicum (o7C) tissues, based on three (_x_) technical replicates of density gradient centrifugation for each sample; DNA concentrations of all samples; mean qPCR standard curve-based estimates of phytoplasma titer for all samples; HTS read mapping results after read down-sampling; and phytoplasma genome assembly quality metrics of down-sampled libraries.

Sample*^a^*	Mean fraction density (g/ml)	DNA con centration (ng/μl)	qPCR *Ct* values	Total reads retained in down-sampling (Million reads)	Mean phytoplasma reads mapped (%)	Mean host gDNA reads mapped (%)	Mean host cpDNA reads mapped (%)	Mean host mtDNA reads mapped (%)	Mean unmapped reads (%)	Phytoplasma genome assembly size (bp)	Largest phytoplasma contig mean (bp)	Phytoplasma genome mean N50 (bp)	Mean reference genome fold coverage
o4P-Homogenate	NA	34.000	16.65	5.0	1.67	85.44	6.48	1.46	4.95	565,143	92,921	37,285	29
o4P-TSE pellet	NA	1.630	12.30	5.0	9.39	69.37	7.57	4.58	9.10	569,323	71,194	28,898	132
**o4P_x_-Fraction1**	**1.049**	**0.668**	**11.87**	**5.0**	**23.37**	**50.80**	**14.48**	**4.04**	**7.31**	**580,942**	**107,718**	**49,236**	**362**
**o4P_x_-Fraction2**	**1.063**	**0.085**	**14.83**	**5.0**	**16.56**	**60.19**	**9.46**	**5.24**	**8.54**	**568,242**	**107,652**	**37,285**	**255**
o4P_x_-Fraction3	1.063	<0.064	16.09	5.0	12.36	61.31	9.05	7.92	9.37	565,250	107,678	37,285	200
**o4P_x_-Fraction4**	**1.078**	**0.214**	**14.50**	**5.0**	**5.69**	**70.08**	**6.99**	**7.81**	**9.43**	**564,351**	**66,839**	**30,110**	**92**
o4P_x_-Fraction5	1.095	<0.094	17.73	5.0	5.00	72.66	5.68	5.87	10.79	566,373	69,878	26,752	92
o4P_x_-Fraction6	1.104	<0.005	19.07	ND	5.74	50.51	2.26	1.29	40.20	565,998	71,194	33,168	60
o4P_x_-Fraction7	1.133	<0.005	19.35	ND	4.06	69.12	2.68	1.44	22.70	565,113	107,877	37,285	57
o4P_x_-Fraction8	1.196	<0.005	21.66	ND	4.24	51.38	2.78	1.90	39.71	566,077	71,194	38,094	54
o7C-Homogenate	NA	61.900	23.80	3.4	0.27	79.33	2.60	2.75	15.05	385,814	3,547	0,936	6
o7C-TSE pellet	NA	17.300	16.58	3.4	0.21	62.42	3.27	3.53	30.57	279,439	3,420	0,723	5
**o7C_x_-Fraction1**	**1.058**	**0.147**	**22.76**	**3.4**	**0.75**	**38.65**	**5.67**	**14.38**	**40.55**	**495,819**	**15,341**	**4,892**	**19**
**o7C_x_-Fraction2**	**1.072**	**<0.005**	**26.46**	**3.4**	**0.57**	**28.53**	**3.96**	**7.52**	**59.43**	**514,099**	**7,684**	**1,943**	**13**
**o7C_x_-Fraction3**	**1.073**	**<0.059**	**26.05**	**3.4**	**0.59**	**31.23**	**2.79**	**7.07**	**58.33**	**507,400**	**7,285**	**1,622**	**12**
**o7C_x_-Fraction4**	**1.083**	**0.860**	**19.38**	**3.4**	**0.43**	**30.40**	**1.25**	**6.75**	**61.18**	**361,156**	**4,497**	**0,981**	**9**
o7C_x_-Fraction5	1.108	<0.198	22.79	3.4	0.24	14.58	1.44	6.36	77.38	341,085	4,040	0,932	7
o7C_x_-Fraction6	1.109	0.057	24.70	3.4	0.41	17.16	1.81	8.39	72.23	463,744	5,106	1,224	9
o7C_x_-Fraction7	1.119	<0.060	23.31	3.4	0.61	30.16	2.03	14.23	52.98	545,833	13,829	2,542	12
o7C_x_-Fraction8	1.157	<0.082	26.23	3.4	0.37	23.31	1.95	8.83	65.54	397,225	5,341	1,008	9

N50 refers to the contig length such that contigs of equal or greater lengths produce 50% of the genome’s length. Note that not all technical replicates were sequenced for all fractions derived from density gradient centrifugation (see Section “Results”).

^a^Sample naming convention: the suffix “-Homogenate” refers to the unprocessed tissue homogenate, “-TSE pellet” refers to the pellet, resuspended in TSE buffer that results after two rounds of differential centrifugation and is loaded to the top of the iodixanol density gradient, “-F1 to -F8” indicates the iodixanol fraction sampled per host.

Bold indicates averages taken from replicates submitted for HTS, values not in bold font indicates a single replicate submitted for HTS.

NA, not available; ND, not done; UD, undetermined.

After performing density gradient centrifugation, the average DNA concentrations for all fractions were below 1 ng/μl regardless of the host and were often below the detectable range of the Qubit™ 1X dsDNA HS Assay Kit (Table 3 and Supplementary Table 2). The average DNA concentrations were highest in Fractions 1 and 4 for the potato replicates (0.67 and 0.21 ng/μl, respectively). For the capsicum replicates, Fraction 4 had the highest average DNA concentration at 0.86 ng/μl, followed by Fraction 5 (0.20 ng/μl) and then Fraction 1 (0.15 ng/μl).

### Evaluations of phytoplasma genome reference assemblies

The draft phytoplasma reference genome assembled from the potato host (Fraction 2 from replicate 2) is 555,927 bp in size (N50 = 41,668 bp) and consists of 30 contigs (Table 2). The average GC content of this genome is 23.9%. The annotation includes 1 16S rRNA gene, 25 tRNA genes, and 463 protein-coding genes. Similarly, the draft phytoplasma reference genome assembled from the capsicum host (Fraction 7 from replicate 1) is 555,321 bp (N50 = 41,719 bp), consists of 29 contigs, with an average GC content of 23.7%. The annotation includes 1 16S rRNA gene, 25 tRNA genes, and 458 protein-coding genes (Table 2). Alignments of the *secA* and *tufB* genes extracted from the reference genomes of strains o4P and o7C with those available for ‘*Ca.* Phytoplasma aurantifolia’ and ‘*Ca.* Phytoplasma australasia’ (Table 2) showed that the phytoplasma strains investigated here were more closely related to ‘*Ca.* Phytoplasma australasia’ (100% nucleotide sequence identity for both genes) than to ‘*Ca.* Phytoplasma aurantifolia’ (91–94% nucleotide sequence identity).

### Evaluations of putative phytoplasma plasmids from reference assemblies

A contig with BLASTn hits to publicly available phytoplasma plasmids were identified for the potato and capsicum hosts investigated in this study. The putative plasmid-derived contigs varied in size for the different hosts, at 4,370 bp for the potato sample with two open reading frames encoded, and 2,913 bp for the capsicum sample encoding three open reading frames. Both putative plasmid contigs contained an approximately 230 bp region bearing >97% sequence identity with the Tomato big bud phytoplasma plasmid (pTBBperi, DQ119297, 3319 bp total length).

### Phytoplasma enrichment evaluations using quantitative polymerase chain reaction and down-sampled high-throughput sequencing sequence data

All negative control samples did not show amplification in the phytoplasma qPCR assays (Supplementary Table 2) and over 98% of their HTS reads remained unmapped after reference-based mapping using the phytoplasma and host genomes as references (Supplementary Table 3). Additionally, the percentages of unmapped, phytoplasma- and host-derived reads of the down-sampled trimmed reads were almost identical to the percentages in corresponding libraries that were not down-sampled (Supplementary Table 3).

#### Potato sample

The qPCR assay for the potato sample plate met the MIQE guidelines (Bustin et al., 2009) with an *R*^2^ value of 0.997 and an efficiency of 99.993%. The starting relative phytoplasma concentration in the DNA extracted from the unprocessed potato homogenate indicated a high phytoplasma titer with a cycle threshold (Ct) of 16.65 (Table 3 and Supplementary Table 2). The estimated phytoplasma concentration increased after the differential centrifugation, with a *Ct* value of 12.30, for this sample. The phytoplasma concentration was also higher than the unprocessed homogenate in Fraction 1 (mean *Ct* of 11.87, range 11.62–12.29), Fraction 2 (mean *Ct* of 14.83; range, 14.42–15.23), Fraction 3 (mean *Ct* of 16.09; range, 15.39–16.79), and Fraction 4 (mean *Ct* of 14.50; range, 14.20–14.90). Therefore, all three technical replicates were prepared and submitted for Illumina sequencing for potato Fraction 1, Fraction 2, and Fraction 4, and only one replicate of the remaining fractions were sequenced.

Mapping results from the total DNA extract from the potato tissue homogenate indicated that the library was composed of 85.44% gDNA, 6.48% cpDNA, and 1.46% mtDNA (a total host-derived DNA of 93.38%, Table 3). Additionally, 1.67% of the reads from the unprocessed tissue homogenate were phytoplasma-derived, and approximately 5.0% of reads remained unmapped (Table 3). After differential centrifugation, the percent of the host-derived reads were reduced to 81.51% (69.37% gDNA, 7.57% cpDNA, and 4.58% mtDNA) of the total library, while the phytoplasma-derived and the unmapped reads increased to 9.39 and 9.10% of the library, respectively. Fraction 1 consistently showed the highest proportion of phytoplasma reads across all three technical replicates (mean, 23.37%; range, 19.12–29.58%). The replicates of Fraction 2 showed the second highest level of phytoplasma reads (mean, 16.56%; range, 14.89–19.04%). The reads mapping to potato gDNA were reduced to an average of 69.32% for Fraction 1, and 74.90% for Fraction 2 (Table 3) compared to the unprocessed tissue homogenate (85.44%). The percent of the cpDNA-derived reads was higher for Fraction 1 (mean, 14.48%; range, 13.09–16.09%), Fraction 2 (mean, 9.46%; range, 9.29–9.51%), and Fraction 3 (7.92%) than in the unprocessed homogenate and differentially centrifuged pellet.

Taxonomic profiling of all unmapped reads indicated that bacteria were enriched for after the differential centrifugation and in the iodixanol fractions showing phytoplasma enrichment when compared to the unprocessed homogenate (e.g., ∼90% bacteria and ∼9% Eukaryota in unprocessed homogenate *vs.* ∼96% bacteria and ∼3% Eukaryota in Fraction 1; Supplementary Figure 1).

When the percent of phytoplasma reads were high as in Fraction 1 after gradient enrichment, the reads spanned the entire potato phytoplasma reference genome (Supplementary Figure 2A) with an average of 362-fold coverage after mapping (Table 3 and Supplementary Table 2) when down-sampled to 5 million reads/library. Libraries with a low percentage of phytoplasma reads left a few regions of the reference genome unmapped and with a lower percentage of overall coverage (e.g., 29-fold coverage for the unprocessed potato homogenate, and 132-fold coverage for the differentially centrifuged pellet; Supplementary Figure 2A).

#### Capsicum sample

The qPCR assay for the capsicum 96-well qPCR plate met the MIQE guidelines (Bustin et al., 2009) with an *R*^2^ value of 0.990 and an efficiency of 101.049%. The *Ct* value for the capsicum unprocessed homogenate was 23.80 based on the qPCR assay (Table 3 and Supplementary Table 2). The phytoplasma concentration increased after differential centrifugation, with a *Ct* value of 16.58 (Table 3 and Supplementary Table 2). Phytoplasma cells were also enriched compared to the unprocessed homogenate after density gradient centrifugation in Fraction 1 (mean *Ct* 22.76; range, 22.25–23.12), Fraction 4 (mean *Ct* 19.38; range, 18.62–19.83). Since phytoplasma cells seemed to be enriched in Fraction 1 and Fraction 4 for the capsicum sample, all three technical replicates for those fractions were prepared and submitted for Illumina sequencing. All three technical replicates for Fraction 2 and Fraction 3 were also sequenced to obtain mapping profiles for all fractions between Fraction 1 and Fraction 4. One technical replicate was sequenced for the remaining fractions.

The mapping results from the total DNA extract from the capsicum tissue homogenate indicated that the library was composed of 84.68% host-derived reads (79.33% gDNA, 2.60% cpDNA, and 2.75% mtDNA), 0.27% phytoplasma-derived reads, with 15.05% of reads remaining unmapped (Table 3 and Supplementary Table 2). After differential centrifugation, the percent of host-derived reads were reduced to 69.22% (62.42% gDNA, 3.27% cpDNA, and 3.53% mtDNA), and phytoplasma-derived reads reduced slightly to 0.21%, while unmapped reads approximately doubled to 30.57% of the library. Fraction 1 showed the highest average enrichment of phytoplasma DNA (mean, 0.75%; range, 0.47–1.04%), followed by the single replicate of Fraction 7 (0.61%) and Fraction 3 (mean, 0.59%; range, 0.37–0.90%). The total percent of capsicum gDNA-derived reads were reduced to below an average of 45% in all iodixanol fractions. The reads derived from the capsicum cpDNA were enriched in the differentially centrifuged pellet, Fraction 1 and Fraction 2 compared to the unprocessed homogenate, but the percent of cpDNA was reduced for the remainder of the iodixanol fractions. Capsicum mtDNA appeared to be enriched with the phytoplasmas, however, and increased from 2.7% in the unprocessed homogenate to over 14.0% in Fraction 1 and Fraction 7.

Taxonomic profiling indicated that the unmapped reads of the capsicum sample were mostly of bacterial origin regardless of the enrichment procedure, at over 99.5%, with differential centrifugation increasing this percentage the most to 99.95% (Supplementary Figure 1).

When the phytoplasma reads were high as in Fraction 1 after iodixanol gradient enrichment, the reads spanned more regions of the capsicum phytoplasma reference genome (Supplementary Figure 2B) with a higher coverage (average of 19-fold coverage after mapping with 3.4 million reads/library, Table 3 and Supplementary Table 2) than those without iodixanol-based enrichment. The samples for which the phytoplasma enrichment was not achieved showed a lower fold-coverage of the reference genome and mapping to fewer regions of the phytoplasma reference genome of the capsicum sample (e.g., five and sixfold coverage of the reference genome from 3.4 million reads for the unprocessed capsicum homogenate and differentially centrifuged pellet samples, respectively, Table 3, Supplementary Figure 2B, and Supplementary Table 2).

### Effect of enrichment on phytoplasma genome assembly quality (down-sampled assemblies)

#### Potato sample

For the potato-derived phytoplasma genomes assembled from 5 million reads, genomes obtained from Fraction 1 were approximately 15,800 bp larger than the assembly from the unprocessed potato homogenate and 11,600 bp larger than the differentially centrifuged pellet. Phytoplasma genomes from Fraction 1 had the highest N50 values (mean, 49,236 bp; range, 28,898–59,408 bp), while the genome N50 values of the unprocessed potato homogenate and the differentially centrifuged pellet genomes were smaller at 37,285 and 28,898 bp, respectively, with the differentially centrifuged pellet showing one of the smallest genome N50 values for all the samples analyzed (Table 3 and Supplementary Table 2). On average, the largest assembled contig was approximately 107,700 bp for Fraction 1, Fraction 2, and Fraction 3, while the largest assembled contig for the unprocessed pellet and differentially centrifuged pellet was 92,900 and 71,200 bp, respectively (Table 3 and Supplementary Table 2).

A putative phytoplasma plasmid was only recovered in one replicate of Fraction 4 for the potato in the down-sampled genome assemblies (data not shown). The putative plasmid was 3,832 bp in size and encoded two open reading frames.

#### Capsicum sample

For phytoplasma genomes assembled from the infected capsicum sample (down-sampled to 3.4 million reads), the largest genomes were assembled for Fraction 7 (545,833 bp), Fraction 2 (mean, 514,099 bp), Fraction 3 (mean, 507,400 bp), and Fraction 1 (mean, 495,819 bp). The phytoplasma genomes assembled from the unprocessed capsicum homogenate and differentially centrifuged pellet were two of the smallest assemblies at 385,814 and 279,439 bp, respectively. Fraction 1 produced a genome assembly with the highest average genome N50 value (mean, 4,892 bp), followed by Fraction 7 (mean, 2,542 bp), and Fraction 2 and Fraction 3 with average genome N50 values of 1,943 and 1,622 bp, respectively (Table 3 and Supplementary Table 2). The genome assemblies from the unprocessed homogenate and differentially centrifuged pellet had similar N50 values, which were below 1,000 bp on average. The largest phytoplasma contig assembled on average was produced for Fraction 1 (mean, 15,341 bp), followed by Fraction 7 (mean, 13,829 bp), Fraction 2 (mean, 7,684 bp), and Fraction 3 (mean, 7,285 bp). The largest contig assembled in the unprocessed homogenate and the differentially centrifuged sample were similar in size at 3,547 and 3,420 bp, respectively.

The putative phytoplasma plasmids were recovered from all replicates of Fraction 1, Fraction 3, Fraction 5, and Fraction 7, as well as from two replicates of Fraction 2. A putative plasmid most similar in size to that recovered for the reference genome assembly was identified from Fraction 3 (technical replicate 2) and was 3,396 bp in size (data not shown).

### Whole genome comparisons

**FIGURE 1:**
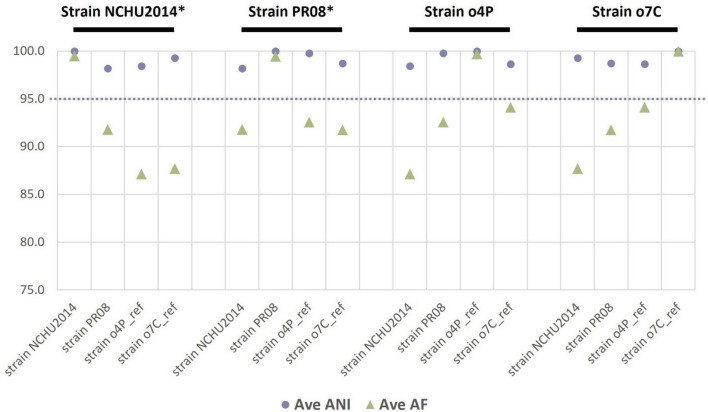
Reciprocal mean average nucleotide identities (ANI, %) and proportion of aligned genomic segments (AF, %) for the two phytoplasma reference genomes assembled in this study and two publicly available phytoplasma reference genomes (strain NCHU2014 and strain PR08). The phytoplasma genome used as a reference in each case is indicated above the graph in black font, with an asterisk indicating a complete genome. The dashed line indicates the proposed 95% ANI species delimitation threshold value.

As an additional estimate of the genetic divergence of the reference genomes (publicly available and those assembled in this study), we calculated the ANI and the proportion of mapped genomic segments between these genomes in a reciprocal manner (Figure 1). The lowest ANI values between two different genomes were between the publicly available reference genomes of strain NCHU2014 and strain PR08 at 98.2%; while the highest ANI was 99.8% between strain NCHU2014 and the ‘*Ca.* Phytoplasma australasia’-related strain o4P. The reference genome of strain o7C shared 94.1% of its genomic segments with the reference genome of strain o4P. The reference genomes of strains o4P and o7C shared 87.1–87.7% of mapped genomic segments with strain NCHU2014, and over 91.8% mapped reads with the publicly available genome of strain PR08.

**FIGURE 2:**
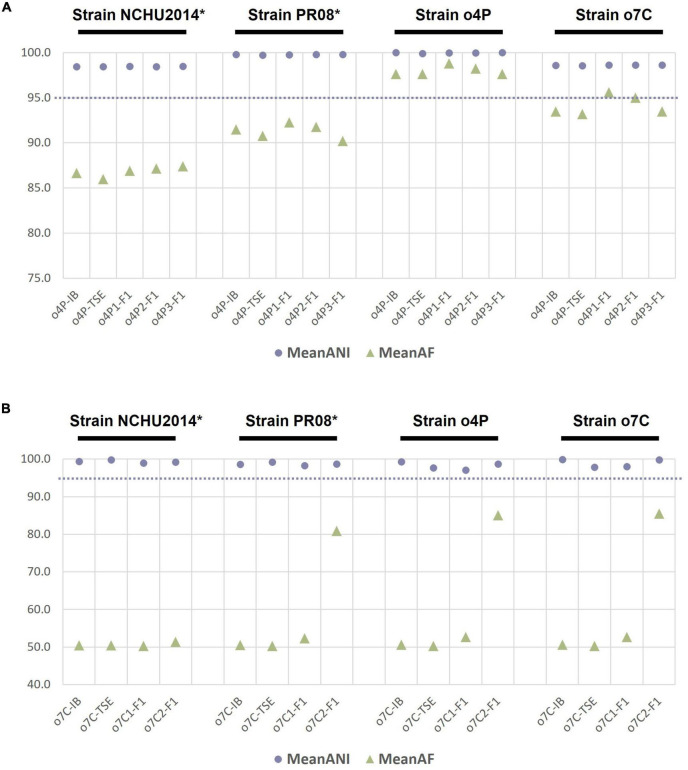
Average nucleotide identity (ANI, %) and proportion of aligned genomic segments (AF,%) for down-sampled assemblies compared to the phytoplasma reference genomes assembled in this study and two publicly available phytoplasma reference genomes for **(A)** the infected potato host and **(B)** the infected capsicum host. The samples analyzed for each host included the unprocessed tissue homogenate (suffix “-IB”), differentially centrifuged pellet (suffix “-TSE”), and the iodixanol fraction with the highest percent of phytoplasma reads (suffix “-F1”). The phytoplasma genome used as the reference in each case is indicated above the graph in black font, with an asterisk indicating a complete genome. The dashed line indicates the proposed 95% ANI species delimitation threshold value.

To evaluate whether genome sequencing of phytoplasma genomes after iodixanol-based enrichment is suitable for comparative genomic analyses, we analyzed the ANIs of genomes from the down-sampled assemblies of the unprocessed homogenate, the differentially centrifuged pellet, and the iodixanol fraction with the highest proportion of phytoplasma-mapped HTS reads of the infected potato and capsicum hosts against the four reference genomes of the closely related taxa used in this study (Figure 2). The ANI values for the phytoplasma genomes assembled from 5 million reads for the unprocessed homogenate (o4P-IB), differentially centrifuged pellet (o4P-TSE), and three replicates of iodixanol Fraction 1 (o4P1-F1 to o4P3-F1) from potato differed by less than 1% in comparisons with all reference genomes used in this study (Figure 2A). The proportion of mapped genomic segments between these genomes ranged between 86.0 and 98.8% (Figure 2A). Iodixanol density gradient centrifugation resulted in a higher proportion of mapped genomic segments compared to the homogenate and differentially centrifuged pellet, but the difference was less than 1% in the case of the infected potato. The ANI values from the capsicum phytoplasma down-sampled assemblies for the unprocessed homogenate (o7C-IB), differentially centrifuged pellet (o7C-TSE), and two replicates of the iodixanol Fraction 1 (o7C1-F1 and o7C2-F1) differed by less than 3% in comparisons with all reference genomes (Figure 2B). The capsicum phytoplasma genomes assembled from iodixanol Fraction 1 consistently had a much greater proportion of mapped genomic segments (52.7–85.5%) with the reference genomes compared to the unprocessed capsicum homogenate and the differentially centrifuged pellet (50.2–50.6%; Figure 2B).

## Discussion

Both qPCR and mapping of HTS reads showed phytoplasma enrichment using the iodixanol density gradient centrifugation method developed in this study. This enrichment, in turn, significantly improved the contiguity and coverage of phytoplasma genome assemblies obtained from the metagenomic data for the two naturally infected phytoplasma hosts investigated here, irrespective of the relative starting titer (Table 3 and Supplementary Figure 2). The highest levels of phytoplasma enrichment were observed in the lower density iodixanol fractions, between an average density of 1.049 and 1.058 g/ml (Fraction 1) and a maximum average density of 1.078–1.083 g/mL (Fraction 4). Host gDNA was also consistently depleted in these iodixanol fractions, shown by at least 30% less HTS reads mapping to the host gDNA reference in the chosen iodixanol fraction than the unprocessed homogenate for both hosts (Table 3). Additionally, we obtained high-quality draft phytoplasma genome sequences from both *Solanaceae* hosts investigated in this study from fractions that had undergone iodixanol density gradient centrifugation (Table 3).

The study demonstrates that iodixanol is ideal to enrich for delicate, wall-less phytoplasma cells due to the non-ionic, non-toxic, and metabolically inert nature of the substance, which help keep cells intact (Ford et al., 1994). Other factors that reduce the risk of cell lysis include the fact that iodixanol is iso-osmotic and maintains a low osmolarity throughout the gradient; thus, preventing pressure-sensitive phytoplasma cells from experiencing large pressure changes during centrifugation. Moreover, iodixanol has a low viscosity allowing for cells to separate based on their buoyant densities during a relatively short centrifugation time, further reducing the risk of lysing phytoplasma cells. Additional precautions to keep the phytoplasma cells intact for their improved recovery included the use of the Phytoplasma Grinding Buffer for cell plasmolysis (Kirkpatrick et al., 1987) which may stabilize phytoplasma cells by reducing their turgor pressure, as well as gentle tissue homogenization and by maintaining all buffers, reagents, tubes, and centrifuges at 4°C throughout the method.

Phytoplasma-derived DNA was detected throughout the density gradient at various proportions for both hosts investigated (Table 3). This may be due to several factors that could affect phytoplasma cell sizes and densities, including their stage in reproduction (parent cell vs. budding cell), their extracellular components (plasmid present or absent, number of plasmids per cell), and the pleiomorphic nature of phytoplasma cells. Cell-free phytoplasma DNA may also separate throughout the iodixanol gradient during centrifugation. These factors and their effect on the efficiency of phytoplasma enrichment by iodixanol density centrifugation, however, remain to be determined.

Phytoplasma genome quality improved significantly with host gDNA depletion using the iodixanol density gradient centrifugation method developed here. We adjusted and tested the effect of the number of differential centrifugation rounds (work by Shrestha, 2021) and the iodixanol gradient set up (data not shown) while developing the method described here, and chose the best of these conditions for this study. However, improvements could be made to further reduce contaminating host gDNA which, based on HTS mapping, was still present at different concentrations throughout the iodixanol gradient after centrifugation (Table 3). This would be particularly important for extremely low-titer hosts. Additional adjustments could be applied to further reduce host gDNA to improve sequencing outputs and phytoplasma genome assembly, including applying additional rounds of differential centrifugation, altering the times and speeds of differential and/or density gradient centrifugation, or even adjusting the concentrations of the isotonic buffer or iodixanol density gradient solutions. Coupling the differential centrifugation step with filtration methods applied in other studies (Palmano, 2001; Tan et al., 2021) could further deplete host cells, and therefore gDNA. However, there is the risk of reducing the phytoplasma yield due to their cell lysis by filtration and/or due to the longer processing time prior to density gradient centrifugation. Another, possibly better-suited approach, involves combining iodixanol density gradient centrifugation with chemical depletion of methylated host gDNA using a commercial kit to the DNA extractions from iodixanol fractions prior to Illumina sequencing. In previous studies, this kit was effective for phytoplasma enrichment by host gDNA reduction with Kirdat et al. (2021) stating a fivefold host gDNA reduction based on host-specific qPCR quantification; and close to 60% less host gDNA reads as identified by their taxonomic classification in Nijo et al. (2021). An issue to applying the kit is the low DNA concentration obtained from the fractions. This could be addressed by resuspending the DNA in a lower volume of eluate (at the expense of their volumes used in the qPCR assay) or by pooling corresponding fractions from multiple replicates. An increase in total DNA would have an additional advantage as it would permit the use of Oxford Nanopore sequencing for long-read sequencing, which has proven useful for improving the contiguity and completeness of phytoplasma genomes (e.g., ‘*Ca.* Phytoplasma aurantifolia’-related strain NCHU2014, Tan et al., 2021; Table 2).

The reduction of host gDNA was consistently observed between Fraction 1 and Fraction 4 of the iodixanol density gradient and greatly improved the quality of phytoplasma genome assemblies (phytoplasma genome assembly sizes up to 137,000 bp larger than without enrichment, N50 values up to 12,000 bp larger, Table 3; and higher read coverage, Supplementary Figure 2). The host-associated non-phytoplasma bacterial DNA, mtDNA, and cpDNA were almost always enriched in these fractions, however, and therefore remain as competitors for HTS sequencing reads (Table 3). This was expected as the plant organelles and bacterial cells are smaller and have a lower density than nuclei (Graham et al., 1994). Additionally, similar iodixanol-based density gradient centrifugation methods have been used to enrich for various subcellular components, such as mitochondria (Choi et al., 2021), as well as Gram-negative bacteria from human and animal cells (Henríquez et al., 2003; Beder et al., 2016) and from insect vectors (Frampton et al., 2018). Similar patterns of host-associated organismal and organellar enrichment was also observed for two phytoplasma enrichment methods recently investigated, one involving methylated DNA depletion (Nijo et al., 2021) and the other developed for phytoplasma-specific enrichment by immunoprecipitation (Tan et al., 2021). Plant organellar DNA “contamination” of phytoplasma-enriched DNA has also been observed for the CsCl density gradient centrifugation method (Malembic-Maher et al., 2008) as these organelles contain AT-rich genomes similar to phytoplasmas (Tran-Nguyen and Schneider, 2012). Improving the specificity of current phytoplasma enrichment methods, combining multiple phytoplasma enrichment and/or host depletion methods, or developing host organellar DNA depletion methods would further enhance the efficiency and therefore the cost of phytoplasma whole genome sequencing in the absence of a phytoplasma culture system. Nevertheless, non-phytoplasma bacterial enrichment by the iodixanol density gradient method developed here is broadly useful, serving as a method that is suitable for genome sequencing of other unculturable and intracellular phytopathogenic bacteria, such as ‘*Candidatus* Liberibacter’ species (Frampton et al., 2018).

The iodixanol density gradient method described here is a significant improvement over some other methods used to enrich for phytoplasmas because it is cost- and time-efficient, and offers increased safety for researchers. For example, during phytoplasma enrichment with CsCl density gradient centrifugation, there are multiple centrifugation steps amounting to over 200 h (Tran-Nguyen and Gibb, 2007), while centrifugation through the iodixanol density gradient in this study only took 3 h. Additionally, the iodixanol density medium is an inert and clinical-grade reagent, making it a much safer to handle compared to those associated with CsCl-based enrichment (Andersen and Liefting, 2013). The iodixanol-based method only made use of (ultra)centrifuges and the appropriate tubes, without the requirement for additional and less frequently used equipment or consumables, making the method more widely feasible and cost-effective for many users to set up. Using the method described here also allowed for direct sequencing from the natural host. This ensures that the most biologically relevant phytoplasma is sequenced and a high-quality genome obtained (Table 2), without requiring transmission to a high-titer experimental host such as periwinkle, which is not always feasible or practical.

The reference genomes of two ‘*Ca.* Phytoplasma australasia’-related strains were assembled from the naturally infected potato and capsicum hosts used in this study, which were all comprised of 30 or fewer contigs and with high contiguity (N50 values of approximately 40,000 bp) (Table 2). This was due to the high sequencing depth achieved for some of the samples coupled with phytoplasma enrichment by iodixanol density gradient centrifugation (see o4P2-F2 and o7C1-F7 in Supplementary Table 2). Since the genomes represent moderately contiguous assemblies, these genomes could be useful in comparisons of whole genome structure and synteny. Their sizes are similar to previous PFGE-based size estimates of closely related phytoplasma genomes, which range between 605,000 and 790,000 bp (Marcone et al., 1999; Padovan et al., 2000). However, it is difficult to estimate true phytoplasma genome sizes and, therefore, the level of completeness of the reference genomes assembled in this study. This is not only due to the tendency of PFGE-based estimates representing an overestimate of genome size by 10–15% (Tsai et al., 2018; Music et al., 2019) but also because natural genome size variations exist even between closely related phytoplasma strains attributed to the high genomic plasticity reported for phytoplasma genomes (Andersen et al., 2013; Music et al., 2019; Huang et al., 2022). Although a high sequencing depth of Illumina reads was achieved, short-read technologies and their associated assembly programs are often unable to resolve the repeat-rich regions of phytoplasma genomes, indicating that there is an opportunity to resolve the gaps in these reference genomes using Sanger and/or long-read sequencing technologies in the future. Moreover, contigs representing putative phytoplasma plasmids were consistently identified from assemblies of both hosts, but require further validation.

This study illustrates the importance of producing high-quality phytoplasma genomes to make accurate comparative genomic and taxonomic inferences. In our comparative genomic analyses, we show that ANI estimates are only slightly affected by the completeness of the phytoplasma genome. For example, the ANI values between all genomes assembled from the potato or capsicum hosts did not differ more than 3%, irrespective of the level of phytoplasma enrichment (Figure 2). This relatively small error in ANI estimation could pose problems for taxa sitting at the borderline of the 95% ANI threshold which was recently recommended to distinguish two separate phytoplasma species (Cho et al., 2020; Bertaccini et al., 2022). However, the proportion of mapped genomic segments implemented with ANI estimates is more severely affected by the completeness of the genomes under consideration, with a lower proportion mapping with more incomplete genomes (Figure 2). The combination of ANI and the proportion of mapped genomic segments are being recognized as important algorithms for robust estimations of taxonomic boundaries of prokaryotes (Barco et al., 2020). In the ANI analysis presented here, phytoplasma genomes assembled from the iodixanol fraction where the highest level of phytoplasma enrichment was observed performed most similarly to the reference genome assembled from the same host, despite read down-sampling prior to assembly, highlighting the benefits of phytoplasma enrichment and the effectiveness of our iodixanol density gradient method on genomic analyses of phytoplasmas (Figures 1, 2). These results are important to consider as phytoplasma research increasingly embraces high-throughput sequencing to delineate species boundaries of these yet-to-be-cultured phytopathogens (Firrao et al., 2013; Cho et al., 2020; Bertaccini et al., 2022).

## Data availability statement

The data presented in this study are deposited in the NCBI repository (https://www.ncbi.nlm.nih.gov/), accession numbers: JALQCT000000000 and JALQCV000000000.

## Author contributions

BRJ participated in the design of the study and carried out the laboratory work, data analysis, interpretation, and drafting the manuscript. CG participated in the collection of samples. FC, BR, CG, and LT-N participated in design of the study and data interpretation and contributed to reviewing and editing the manuscript. All authors read and approved the final manuscript.

## Footnotes

1
https://github.com/lh3/seqtk

2
www.geneious.com
