# Supplementary material for: Iodixanol density gradients as an effective phytoplasma enrichment approach to improve genome sequencing
Source: Front Microbiol. 2022 Aug 12;13:937648. doi: 10.3389/fmicb.2022.937648 (PMC9411968; doi:10.3389/fmicb.2022.937648)
Supplement: Supplementary file 1 [file Data_Sheet_1.docx]

# Supplementary figures and tables:

**Supplementary Table S1:** Summary of volumes of iodixanol sampled per fraction of the gradient and their mean densities after centrifugation of two iodixanol gradients overlaid with cell-free TSE buffer as a negative control (NO) or overlaid with a preparation of phytoplasma-infected potato (o4P) and capsicum (o7C) tissues, based on two (_y_) or three (_x_) technical replicates of each density gradient for each sample.

| **Sample** | **Average volume per fraction (mL)** | | **Ave density per fraction (g/mL)** | **Min Density (g/mL)** | **Max Density (g/mL)** | **Density standard deviation per fraction across replicates** |  |
| --- | --- | --- | --- | --- | --- | --- | --- |
| o4Px-Fraction1 | | 1.400 | 1.049 | 1.044 | 1.053 | 0.004 |  |
| o4Px-Fraction2 | | 1.400 | 1.063 | 1.059 | 1.068 | 0.004 |  |
| o4Px-Fraction3 | | 1.400 | 1.063 | 1.063 | 1.064 | 0.000 |  |
| o4Px-Fraction4 | | 1.400 | 1.078 | 1.075 | 1.083 | 0.004 |  |
| o4Px-Fraction5 | | 1.400 | 1.095 | 1.093 | 1.097 | 0.002 |  |
| o4Px-Fraction6 | | 1.400 | 1.104 | 1.098 | 1.108 | 0.004 |  |
| o4Px-Fraction7 | | 1.400 | 1.133 | 1.129 | 1.137 | 0.003 |  |
| o4Px-Fraction8 | | 1.987 | 1.196 | 1.187 | 1.210 | 0.010 |  |
| o7Cx-Fraction1 | | 1.400 | 1.058 | 1.057 | 1.059 | 0.001 |  |
| o7Cx-Fraction2 | | 1.400 | 1.072 | 1.067 | 1.078 | 0.004 |  |
| o7Cx-Fraction3 | | 1.400 | 1.073 | 1.068 | 1.078 | 0.004 |  |
| o7Cx-Fraction4 | | 1.400 | 1.083 | 1.067 | 1.094 | 0.011 |  |
| o7Cx-Fraction5 | | 1.400 | 1.108 | 1.094 | 1.117 | 0.010 |  |
| o7Cx-Fraction6 | | 1.400 | 1.109 | 1.105 | 1.117 | 0.005 |  |
| o7Cx-Fraction7 | | 1.400 | 1.119 | 1.116 | 1.123 | 0.003 |  |
| o7Cx-Fraction8 | | 1.993 | 1.157 | 1.088 | 1.193 | 0.049 |  |
| NOy-Fraction1 | | 1.400 | 1.468 | 1.042 | 1.058 | 0.007 |  |
| NOy-Fraction2 | | 1.400 | 1.493 | 1.065 | 1.067 | 0.001 |  |
| NOy-Fraction3 | | 1.400 | 1.494 | 1.059 | 1.071 | 0.006 |  |
| NOy-Fraction4 | | 1.400 | 1.508 | 1.063 | 1.091 | 0.012 |  |
| NOy-Fraction5 | | 1.400 | 1.544 | 1.102 | 1.104 | 0.001 |  |
| NOy-Fraction6 | | 1.400 | 1.550 | 1.104 | 1.111 | 0.003 |  |
| NOy-Fraction7 | | 1.400 | 1.572 | 1.122 | 1.125 | 0.001 |  |
| NOy-Fraction8 | | 1.932 | 2.303 | 1.181 | 1.203 | 0.009 |  |

**Supplementary Figure S1:** Taxonomic classification to the domain-level of unmapped reads (after reference mapping to host and phytoplasma genomes) of non-normalised libraries from the unprocessed homogenate (suffix “-Hom”), differentially centrifuged pellet (suffix “-TSE”), and all successfully sequenced replicates of fractions with the highest phytoplasma concentration for each host (suffix “-F1” for “Fraction 1” or “-F4” for “Fraction 4”) including potato (o4P) and capsicum (o7C). Taxonomic classification was determined using Kraken2. Note that the y-axis starts at 70% for better visualisation of lower abundance domains.

(A)
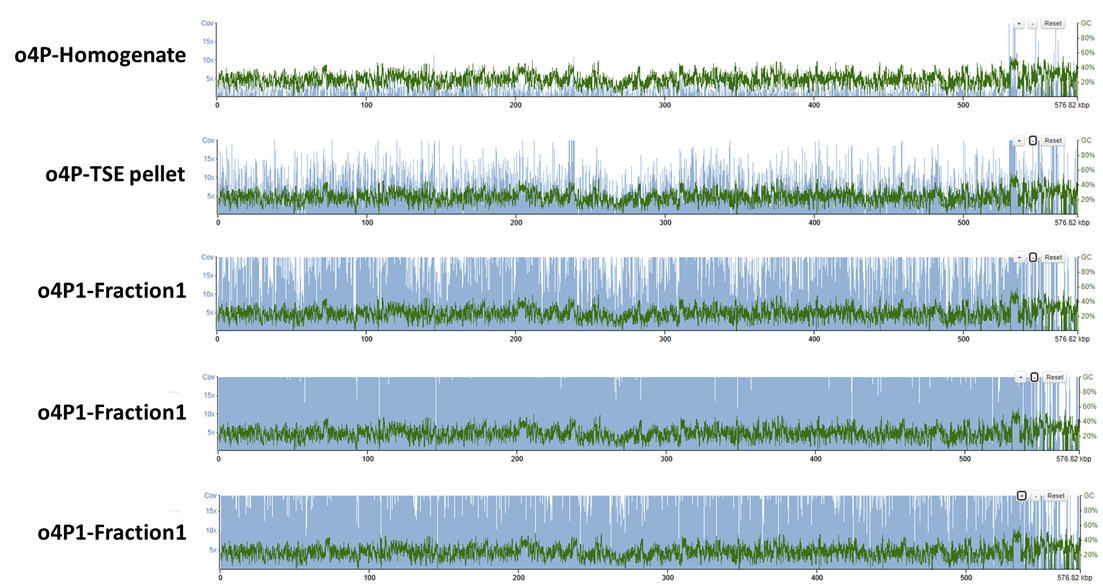


(B)


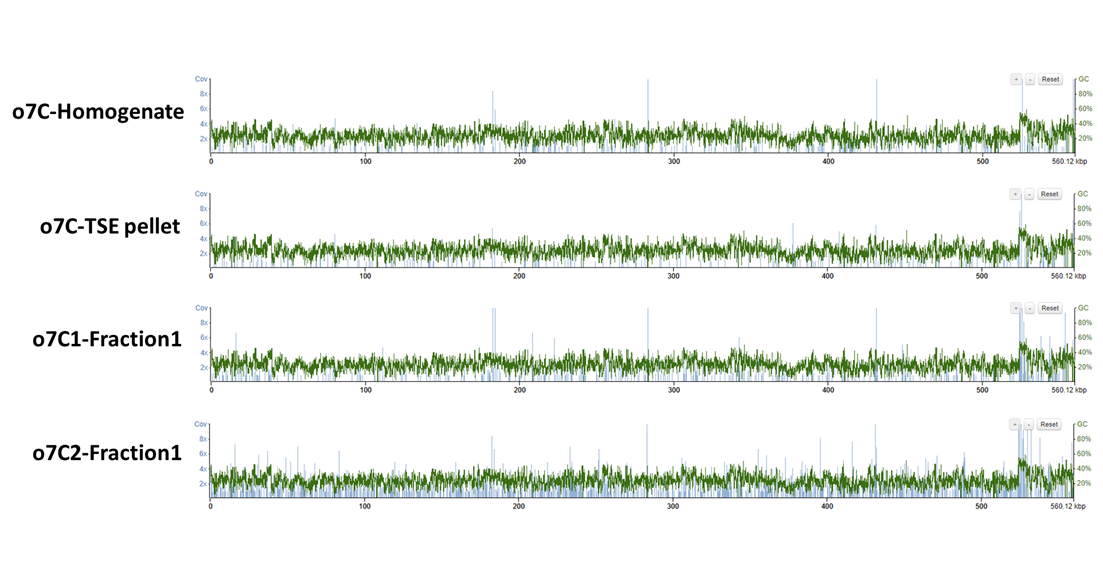


**Supplementary Figure S2:** Visualisation of mapping results for down-sampled sequence reads from the unprocessed tissue homogenate, differentially centrifuged pellet, and highest phytoplasma concentration iodixanol density fraction sampled for phytoplasma infected potato (o4P) and capsicum (o7C). Panel **(A)** indicates results for the phytoplasma potato tissues and its related samples at 5 million reads per library, and panel **(B)** indicates results for the phytoplasma isolated from capsicum tissues and related samples at 3.4 million reads per library. The reference genome is indicated by a solid black line in order of largest contig to the shortest (contig edges are not shown) with its GC percent (%) at each position indicated in dark green (scale on the right of each image in dark green font), and light blue vertical bars represent reads mapping to the reference genome with the scale of read coverage (times coverage, x) indicated on the left of each image in light blue font.
