# Supplementary material for: Iodixanol density gradients as an effective phytoplasma enrichment approach to improve genome sequencing
Source: Front Microbiol. 2022 Aug 12;13:937648. doi: 10.3389/fmicb.2022.937648 (PMC9411968; doi:10.3389/fmicb.2022.937648)
Supplement: Supplementary file 3 [file Table_3.DOCX]

**Appendix File 1**

**Protocol: Sample preparation, differential centrifugation and iodixanol density gradient centrifugation (triple replicates per sample, 1 negative control), DNA extraction**

1. **Materials:**
   1. **Consumables:**
   - UltraPure™ DNase/RNase-Free Distilled Water (Invitrogen™)
   - Iodixanol (Optiprep, Sigma)
   - Chloroform:isoamyl alcohol (24:1)
   - Isopropanol (kept at -20°C)
   - RNase
   - Universal-long 15x28 cm extraction bags (BIOREBA)
   - 50 mL conical screw top tubes
   - 15 mL conical screw top tubes
   - 20 mL sterile syringes
   - 20-gauge needles
   - 13.2 mL open-top thin wall polypropylene centrifuge tubes (Product no. 331372, Beckman Coulter)
   - 1 mL sterile disposable pipette tips
   - 5 mL and 50 mL tip cones compatible with an electronic pipette
   - Sterile scalpels and blades
   - Ice
   - Qubit™ 1X dsDNA HS Assay Kit (Invitrogen)
   1. **Reagents to prepare:**
   - Phytoplasma Grinding Buffer (100 mM K_2_HPO_4_, 30 mM KH_2_PO_4_, 10% sucrose, 2% polyvinylpyrrolidone-10. On day of use, add 0.15% bovine serum albumin, 25 mM ascorbic acid; pH 7.6 at 4°C)
   - Iodixanol diluent solution (0.15 M NaCl, 1 mM EDTA, 15 mM Tris; pH 7.6 at 4°C)
   - Iodixanol working solution diluent (0.15 M NaCl, 0.5 mM EDTA, 5 mM Tris; pH 7.6 at 4°C)
   - Tris-Sucrose-EDTA (TSE) buffer (200 mM Tris–HCl, pH 8.0, 500 mM sucrose, 1 mM EDTA; pH 8.0 at 4°C)
   - Freshly prepared 75% ethanol
   1. **Equipment:**
   - Analytical balance measuring up to three decimal places
   - Bench top centrifuge fitted with the fixed-angle F-3-6-38 rotor (Eppendorf)
   - Bench top centrifuge fitted with the swinging bucket A-4-44 rotor (Eppendorf)
   - Optima L-100 XP ultracentrifuge (Beckman Coulter) fitted with a SW 41 Ti rotor (Beckman Coulter)
   - Benchtop vortex
   - Biosafety cabinet
   - Electronic pipette compatible with 5 mL and 50 mL tip cones
   - Fridge set at 4°C
   - Homex tissue grinder
   - Icebox
   - pH meter
   - Qubit™ 2.0 fluorometer (Invitrogen)
   - Variable volume single channel pipette, 100-1000 µL
2. **Procedure:**


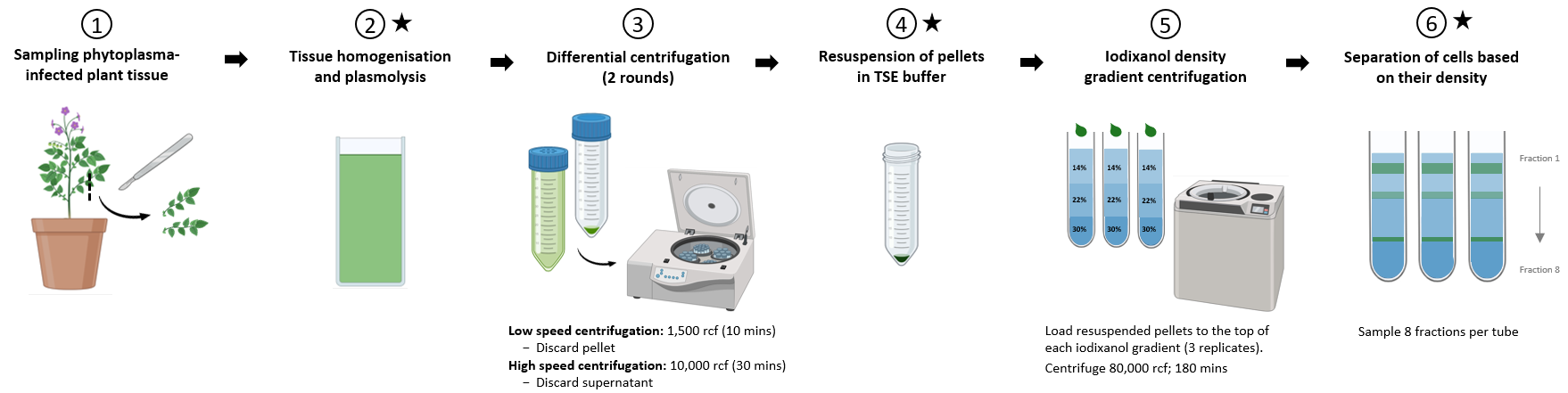


**Appendix Figure A1:** Workflow of the iodixanol-based phytoplasma enrichment method developed in this study. The workflow begins at (1) plant material sampling and is followed by (2) homogenisation, (3) two rounds of differential centrifugation, (4) pellet resuspension, (5) iodixanol density gradient centrifugation, and (6) iodixanol fraction sampling, in that order. Iodixanol fractions are sampled according to Appendix Figure A2. A temperature of 4°C is maintained for all reagents, buffers, and equipment at all steps. Points at which aliquots are sampled for DNA extraction are indicated with . Thereafter, DNA extracts are used in qPCR and metagenomic high throughput sequencing library preparation. Created with BioRender.com.

- 1. **Sample preparation and differential centrifugation (see Appendix Figure A1)**
     1. Pre-cool the bench top centrifuge fitted with the fixed-angle F-3-6-38 rotor (Eppendorf) to 4°C.
     2. Using a scalpel, cut 16 g of plant material from the symptomatic sample and place in a BIOREBA extraction bag, then add 210 mL ice-cold Phytoplasma Grinding Buffer.
     3. Gently grind the Phytoplasma Grinding Buffer-submerged material using a Homex tissue homogeniser at setting 10 of 100.
     4. Place the green homogenate in the fridge for 20 min to allow cells to plasmolyse.
     5. Pour 48 ml of the green homogenate into each of the two ice-cold 50 mL conical screw cap tubes.
     6. Sample an additional 2 mL of green homogenate from the BIOREBA bag and store in a 15 mL centrifuge tube, at -20°C, until the DNA extraction stage. *(Suggested sample name: ‘unprocessed homogenate’)*.
     7. Centrifuge the homogenates in the 50mL conical screw cap tubes at 1,500 rcf in a fixed angle rotor for 10 min at 4°C.
     8. Transfer the resultant supernatants to two new ice-cold 50 mL conical screw cap tubes, discard the pellets, and centrifuge the supernatants at 10,000 rcf for 30 min at 4°C.
     9. Discard the supernatant.
     10. Gently resuspend the pellet in 40 mL TSE buffer using a scraping motion with a 1 mL pipette tip and gentle tipping of the tube. Avoid multiple and rapid aspiration.
     11. Repeat steps 2.1.7 to 2.1.9.
     12. Resuspend the two pellets in 800 µL ice cold TSE buffer using a scraping motion with a 1 mL pipette tip and gentle swirling of the tube. Avoid multiple and rapid aspiration.
     13. Combine the two resuspended pellets into one of the 50 mL conical tubes. Keep on ice in an ice bucket while preparing the next step.
  2. **Iodixanol dilutions (see Appendix Figure A1)**
     1. To make a 40% iodixanol working solution, dilute 30.5 mL of the 60% iodixanol (Optiprep, Sigma) stock solution with 15.25 mL of iodixanol diluent solution in a 50 mL conical screw top tube.
     2. In three separate 50 mL conical screw top tubes, prepare the three different iodixanol dilutions and refrigerate at 4°C until use:
  - Prepare the 14% iodixanol solution by adding 10.5 mL of the 40% iodixanol working solution to 19.5 mL of the iodixanol working solution diluent.
  - Prepare the 22% iodixanol solution by adding: 16.5 mL of the 40% iodixanol working solution to 13.5 mL of the iodixanol working solution diluent.
  - Prepare the 30% iodixanol solution by adding: 16.5 mL of the 40% iodixanol solution + 5.5 mL of the iodixanol working solution diluent
  1. **Iodixanol density gradient set up and centrifugation (see Appendix Figure A1)**

**Note:** keep iodixanol dilutions on ice and mix them well by inversion before setting up the gradients in the ultracentrifuge tubes.

- - 1. Pre-cool the Optima L-100 XP ultracentrifuge (Beckman Coulter) fitted with a SW 41 Ti rotor (Beckman Coulter) to 4°C.
    2. Place a small ice bucket filled with ice in a biosafety cabinet. Place four 13.2 mL open-top thin wall polypropylene centrifuge tubes (Beckman Coulter) into the ice (50 - 75% of the tube should be submerged in ice).
    3. Carefully set up the iodixanol gradient in four 13.2 mL open-top thin wall polypropylene centrifuge tubes (Beckman Coulter) using a 20 mL syringe fitted with a 20-gauge needle:
       1. Add 3 mL of the ice-cold 30% solution into each ultracentrifuge tube.
       2. Overlay 4 mL of the ice-cold 22% solution on top of the 30% solution slowly by letting the solution drip down the side of the tube.
       3. Overlay 4 mL of the 14% solution slowly on top of the 22% solution in the same way.
    4. Overlay 400 µL of the cooled green TSE buffer-suspended pellet onto the top of three of the four iodixanol gradients using a 1 mL pipette. Add 400 µL cell-free TSE buffer to the fourth polypropylene centrifuge tubes. Reserve 400 µL of green pellet in a 2 mL capped centrifuge tube. *(Suggested sample name: ‘differentially centrifuged pellet’).*
    5. Place the polypropylene centrifuge tubes containing the iodixanol gradients and samples in the cooled, metal centrifuge tube holders and replace their caps. Weigh each centrifuge tube in the tube holder and with their caps replaced. Balance tubes to three decimal places using TSE buffer and a 20 µL pipette and tips. Replace tips after every weight adjustment.
    6. Centrifuge the balanced tubes for 180 mins in the precooled Optima L-100 XP ultracentrifuge (Beckman Coulter) at 80 000 rcf in a swinging bucket SW 41 Ti rotor at 4°C.
    7. In the biosafety cabinet, remove the propylene centrifuge tubes and place them on ice.
    8. Using a 1 mL pipette, remove fractions of the iodixanol gradient down the tube (see **Appendix Figure A2** below) and add to a 15 mL screw cap conical tube. Change the pipette tip before sampling the next fraction.
    9. To estimate the density of the sampled fractions (g/mL), zero a balance using an empty screw cap conical tube of the same size and brand, then weigh each sample to 3 decimal places.
    10. Freeze the sampled fractions at -20°C until DNA extraction.


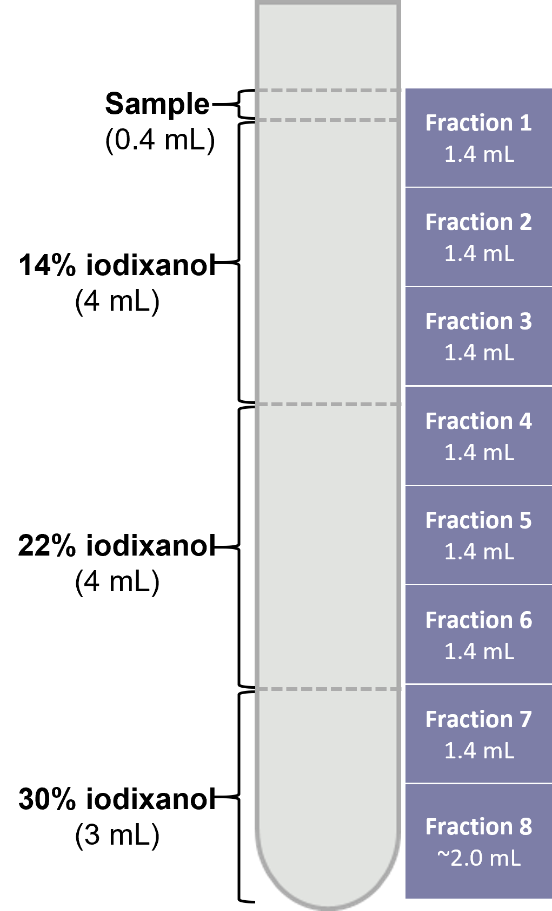


**Appendix Figure A2:** Illustration of the 13.2 mL polypropylene centrifuge tube indicating the volumes of iodixanol dilutions and sample added (left) to create the density gradient prior to centrifugation and the volumes of the fractions of the gradient sampled after centrifugation (right) for a single technical replicate. Fractions are sampled from the top of the tube (Fraction 1) and ending with the last fraction at the bottom of the tube (Fraction 8).

- 1. **DNA extraction for use in library preparation and quantitative PCR (qPCR)**
     1. To each homogenate, pellet, or fraction, add 5 mL of 2.5% CTAB and 10 µL of RNase and vortex/invert the tube for 10 s.
     2. Incubate at 64°C for 20 min and invert the tube every 5 to 10 min (2 to 3 times in total).
     3. Slowly add 5 mL chloroform:isopropyl alcohol (24:1) using an electronic pipette and vortex/invert the tube for 10s.
     4. Centrifuge at 3,500 rcf in a benchtop centrifuge fitted with the swinging bucket A-4-44 rotor (Eppendorf) for 5 mins. During this time, fill fresh, labelled 15 mL conical screw cap tubes with chloroform:isopropyl alcohol (24:1) using an electronic pipette.
     5. Transfer the upper aqueous phase of the centrifuged homogenate, pellet, or fractions using a 1 mL pipette to the new, 15 mL conical screw cap tube filled with chloroform:isopropyl alcohol (24:1). (*Note: Avoid pipetting the white interface and lower aqueous phase with the upper aqueous phase, and change the pipette tip between different samples*).
     6. Repeat steps 2.4.4 – 2.4.5 until no white interface, between the upper and lower aqueous phases, is visible.
     7. Add 5 mL ice cold isopropanol to the final upper aqueous phase of the centrifuged homogenate or fractions and incubate at -20°C overnight.
     8. Centrifuge in the benchtop centrifuge fitted with the swinging bucket A-4-44 rotor (Eppendorf) at 4,500 rcf for 20 mins at 4°C. Slowly decant and discard the supernatant *(Note: take care when decanting as the pellet might not be visible).*
     9. Wash pellet with 5 mL ice-cold, freshly prepared 75% ethanol by centrifugation at 4,500 rcf for 10 mins at 4°C in a benchtop centrifuge fitted with the swinging bucket A-4-44 rotor. Repeat this step once.
     10. Air dry overnight at room temperature in a biosafety cabinet.
     11. Warm UltraPure™ DNase/RNase-Free Distilled Water (Invitrogen™) to 65°C, then resuspend the pellet in 60 µL of the warmed DNase/RNase-Free distilled water.
     12. Measure the concentration of each DNA extract using Qubit™ 1X dsDNA HS Assay Kit (Invitrogen) on a Qubit™ 2.0 fluorometer (Invitrogen), or similar, following manufacturer protocols.
     13. DNA can be used immediately or stored at -20°C until the qPCR and library preparation step.
